# The Impact of Dietary Folate Intake on Reproductive Function in Premenopausal Women: A Prospective Cohort Study

**DOI:** 10.1371/journal.pone.0046276

**Published:** 2012-09-26

**Authors:** Audrey J. Gaskins, Sunni L. Mumford, Jorge E. Chavarro, Cuilin Zhang, Anna Z. Pollack, Jean Wactawski-Wende, Neil J. Perkins, Enrique F. Schisterman

**Affiliations:** 1 Division of Epidemiology, Statistics, and Prevention Research, Eunice Kennedy Shriver National Institute of Child Health and Human Development, Rockville, Maryland, United States of America; 2 Department of Nutrition, Harvard School of Public Health, Boston, Massachusetts, United States of America; 3 Department of Epidemiology, Harvard School of Public Health, Boston, Massachusetts, United States of America; 4 Department of Medicine, Channing Division of Network Medicine, Brigham and Women’s Hospital and Harvard Medical School, Boston, Massachusetts, United States of America; 5 Department of Social and Preventive Medicine, University at Buffalo, The State University of New York, Buffalo, New York, United States of America; University of Washington, United States of America

## Abstract

**Background:**

Folic acid is recommended to reproductive-aged women to prevent birth defects, though little is known about the effects of dietary intake on other reproductive outcomes. Improved pregnancy rates have been documented after folic acid supplement use, suggesting a possible link with ovulation, however research is limited. Our objective was to evaluate the association between dietary folate intake, hormone levels, and sporadic anovulation in healthy, regularly menstruating women.

**Methodology/Principal Findings:**

The BioCycle study (2005–2007) prospectively followed 259 healthy women aged 18–44 years from the western New York region for up to 2 menstrual cycles. Total folate and specific sources of folate were assessed up to 4 times per cycle by 24-hour recall. Estradiol, progesterone, luteinizing hormone, and follicle-stimulating hormone were measured in serum up to 8 times per cycle, timed using fertility monitors. Anovulation was defined as a cycle with peak progesterone concentration ≤5 ng/mL and no LH peak in the mid/late luteal phase. Higher intake of dietary folate (in dietary equivalents) across tertiles had a marginally significant association with greater luteal progesterone levels (*P* trend 0.08). Higher intake of synthetic folate was significantly associated with higher luteal progesterone levels (*P* trend 0.05). Specifically, women in the 3^rd^ tertile of synthetic folate intake had, on average, 16.0% (95% CI, 0.5–33.8%) higher luteal progesterone levels compared to women in the 1^st^ tertile. Moreover, consumption of synthetic folate was significantly and inversely associated with anovulation such that women in the 3^rd^ tertile had a 64% (95% CI, 8–86%) decreased odds of anovulation compared to the women in the 1^st^ tertile (*P* trend 0.03).

**Conclusions/Significance:**

These findings suggest that a diet high in synthetic folate may be associated with increased progesterone levels and lower risk of sporadic anovulation. Further study of the effect of dietary folate and folic acid supplement use on reproductive health is warranted.

## Introduction

In the early 1990s, periconceptional folic acid supplement use was found to reduce both the occurrence [1] and reoccurrence [2] of neural tube defects. These discoveries led not only to the recommendation of folic acid supplement use to reproductive-aged women, but also to the fortification of foods with folic acid in several countries [3], [4], [5], [6]. Despite folic acid supplement use being heralded as one of the most significant public health measures for the prevention of neural tube defects, little is known about the effects of dietary intake on pre-pregnancy reproductive outcomes.

Folate is necessary for the synthesis of DNA, transfer RNA, and the amino acids cysteine and methionine. Thus, it plays an important role in human reproduction [7]. In animals, folic acid supplementation increased ovulation rates (and subsequently litter sizes) in pigs [8] while folic acid deficiency decreased ovulation in rats [9]. Previous human studies documented higher pregnancy rates among users of micronutrient supplements either with [10] or without [11] fertility disorders. Further, regular use of multivitamins was associated with a decreased risk of ovulatory infertility with B vitamins, in particular folic acid, explaining some of this association [12]. However, human studies have yet to pinpoint a specific nutrient in multivitamins explaining the beneficial effect.

The objective of this study was to evaluate the association between dietary folate consumption and reproductive hormone levels and risk of incident sporadic anovulation in the BioCycle Study. Based on folate’s biological activities, we initially hypothesized that a low intake of folate (and subsequent elevated homocysteine accumulation) could lead to reduced cell division, increased inflammatory cytokine production, altered nitric oxide metabolism, increased oxidative stress, elevated apoptosis, and disturbed methylation reactions which could subsequently affect oocyte development [13]. Therefore, our hypothesis was that higher consumption of dietary folate would be associated with higher reproductive hormone concentrations (estrogen, progesterone, luteinizing hormone, and follicle-stimulating hormone) and a lower risk of incident anovulation.

## Methods

The BioCycle Study is a prospective cohort study of menstrual cycle function conducted between 2005–2007 among 259 healthy, premenopausal women 18 to 44 years of age from western New York, and followed for one (n = 9) or two (n = 250) menstrual cycles. To be eligible for the study, women had to have a self-reported cycle length between 21 and 35 days for the past 6 months. Exclusion criteria included a history of gynecological problems, endometriosis, fibroids, or polycystic ovary syndrome, current regular use of oral contraceptives, vitamin and mineral supplements, or prescription medications; pregnancy or breastfeeding in the past 6 months; diagnosis of chronic medical conditions, including metabolic disorders and gastrointestinal diseases associated with malabsorption; self-reported body mass index (BMI) at screening less than 18 or greater than 35 kg/m^2^; or current dietary restrictions for weight loss or medical reasons. Details of the study have been published elsewhere [14]. The University at Buffalo Health Sciences Institutional Review Board (IRB) approved the study, and served as the IRB designated by the National Institutes of Health for this study under a reliance agreement. All participants provided written informed consent.

Fasting blood specimen collection was scheduled in the morning at up to 8 clinic visits per cycle. Visits were timed using fertility monitors (Clear Blue Easy Fertility Monitor; Inverness Medical, Waltham, MA, USA) and occurred on approximately the 2nd day of menstruation, mid and late follicular phase, two days around expected ovulation, and early, mid, and late luteal phase [15]. Collection and handling protocols were designed to minimize variability in pre-analytical factors and have been previously described [16]. All samples were processed and frozen at −80°C within 90 minutes of phlebotomy, later shipped on dry ice to analytical laboratories as a complete cycle, and measured simultaneously, within a single analytical run, to limit analytical variability. 94% of participants completed ≥7 clinic visits per cycle and 100% completed 5 visits per cycle, with fewer visits typically a result of shorter cycles.

Dietary intake was assessed by 24-hour dietary recalls on the same days as blood sample collection and was conducted up to 4 times per cycle (corresponding to menses, mid follicular phase, ovulation, and mid luteal phase), for a total of 8 possible recalls over 2 cycles. The 24-hour recalls were conducted by trained research staff. Dietary data were analyzed using the Nutrition Data System for Research software version 2005 developed by the Nutrition Coordinating Center of the University of Minnesota, Minneapolis, MN. This program computed the nutrients, food components, and food sources from the 24-hour dietary recalls. Intakes of total and specific types of folate per woman were calculated as the sum of the contributions from all foods on the basis of U.S. Department of Agriculture food composition data. Dietary folate equivalents (DFE) (in µg/day) were calculated to account for differences in absorption between natural folate and synthetic folate using the formula: natural folate + (synthetic folate x 1.7) [17]. The majority of cycles had 4 dietary recalls (87%) and all cycles had at least 2 recalls.

Reproductive hormones were measured in fasting serum samples collected at each cycle visit at the Kaleida Health Center for Laboratory Medicine (Buffalo, NY). Estradiol concentrations were measured by radioimmunoassay. Follicle-stimulating hormone (FSH), luteinizing hormone (LH), and progesterone were measured using solid-phase competitive chemiluminescent enzymatic immunoassays by Specialty Laboratories Inc (Valencia, CA) on the DPC Immulite 2000 analyzer (Siemens Medical Solutions Diagnostics, Deerfield, IL). Across the study period the total coefficient of variation (CV) for these tests reported by the laboratory were <10% for estradiol, <5% for LH and FSH, and <14% for progesterone. Cycles with progesterone concentrations ≤5 ng/mL and no observed serum LH peak during the mid or late luteal phase were considered anovulatory cycles [18]. Based on this algorithm, 42 of the 509 cycles (8.3%) in this study were classified as anovulatory. To account for variability in cycle and phase length and potential for mistimed sample collection, visits from ovulatory cycles were realigned based on the date of the LH surge as indicated by the monitors [19]. Anovulatory cycles, as defined above, are included in the current analysis and were not modified by the realignment algorithm.

At baseline, height (in meters) and weight (in kilograms) were measured using standardized techniques and BMI (kg/m^2^) was calculated. Participants also completed questionnaires on physical activity, lifestyle, and reproductive health history. High, moderate, and low physical activity categories were created based on standard International Physical Activity Questionnaire cut points [20]. Women completed diaries on their daily alcohol intake. Based on these responses, women who consumed more than 4 drinks in one setting during a given menstrual cycle were classified as periodic high alcohol consumers. Cycle length was defined as the number of days between menstrual bleeding. Day one of the cycle was defined as menstruating by 4∶00 pm on that day; the last day of the cycle was the last day before the next onset of bleeding. All covariates assessed had at least a 95% response rate.

### Statistical Analysis

Repeated-measures analysis of variance was used to compare intake of dietary folate and other dietary components across the cycle to evaluate patterns of occasional versus habitual folate consumption. As no significant differences were observed, the average daily intake for dietary folate and other dietary variables were calculated per cycle for this analysis. Descriptive statistics were calculated for demographic characteristics and dietary nutrients according to tertile of average dietary folate intake (in dietary equivalents) per day. Generalized linear mixed models were used to test for associations between demographic variables, dietary nutrients and tertiles of dietary folate intake. All comparisons take repeated measures and correlations between cycles into account and p-values presented are two-sided and α = 0.05. Dietary nutrients except for total calories and % of calories from carbohydrate, protein, and fat are adjusted for total calorie intake. Alternative cutoffs were explored to assess sensitivity of results to this categorization, including quartiles and quintiles of folate intake and categories corresponding to the US dietary reference value (400 µg/day of DFE). Dietary composition was also evaluated and the intake of specific types of folate (natural, synthetic, and food sources of folate). Due to the high number of non-consumers of beans, bean folate intake was categorized as non-consumers (0 µg/day), consumers below the median (0.1–81.4 µg/day), and consumers above the median (>81.4 µg/day).

To assess the association between folate intake and reproductive hormone levels, we estimated the effects applying linear mixed models with inverse probability weights [21], [22]. All linear mixed models included random intercepts to account for the variability in baseline hormone levels between women and for the correlation between cycles of the same woman. Inverse probability weights were used to control for confounding because other reproductive hormones (e.g., LH, progesterone, and FSH) represent time-varying confounders that also act as intermediates when investigating the independent association between dietary folate and estrogen. Weights were based on the probability of an individual consuming the amount of folate they actually consumed, conditional on their fixed (demographic and dietary characteristics) and time-varying (current and past hormone levels) covariates. Ordinary least-squares regression was employed to obtain the predicted probabilities used in constructing the stabilized weights.

Reproductive hormone levels were log transformed for normality and results are presented as percent change from the reference category, (exp(β)−1)×100. For models of estradiol, up to 8 measurements across the cycle were considered; however for progesterone only up to 3 measurements in the luteal phase were considered and for LH and FSH only up to 3 measurements around ovulation were considered. Generalized linear mixed models were used to model the association between average folate intake and the odds of anovulation [23]. The median value of each tertile of dietary folate intake was used as a continuous variable to test for linear trends across tertiles.

The presence of confounding was evaluated using a hybrid approach combining prior knowledge using directed acyclic graphs (DAGs) and a statistical approach based on change in point estimates [24]. A set of variables was determined by a review of the prior literature and a detailed DAG was created that identified the variables that should be included in the models. Covariates were then included in the model if they changed the exposure coefficient by more than 15% and were significant at the *P* = 0.10 level. Factors that were found to affect the point estimates were energy intake (continuous), race (white, black, and other), age (continuous), and dietary fiber (continuous).

Highly correlated variables such as iron and vitamins B1, B2, B3, B6, and B12 were analyzed as collinear variables and potential confounders by adding the nutrients to the fully adjusted model separately and then in combination to see if it affected the magnitude and/or significance of the effect estimate for folate. In addition, alternate definitions of anovulation were used to assess the sensitivity of this classification. Effect modification by factors previously documented to affect serum folate levels such as BMI, smoking status, vitamin B12 intake, and alcohol intake (periodic high alcohol intake and average intake per day) were tested using cross-product terms in the final multivariate models (*P* value for significance, 0.10). Further sensitivity analyses included, a stratified analysis of folate and reproductive hormones in ovulatory and anovulatory cycles separately and an analysis using continuous folate (as opposed to categorical folate) as the main exposure. SAS version 9.2 (SAS Institute, Cary, NC, USA) was used for all statistical analyses. All statistical analyses took multiple cycles per woman into account and all p-values presented are two-sided.

## Results

Overall this cohort of women was young (mean age: 27.3 yrs, standard deviation (SD) 8.2) and of normal weight (mean BMI: 24.1 kg/m^2^, SD 3.9). The majority of these women were physically active (90.8% moderate to high physical activity), had never smoked (81.9%), and were nulliparous (73.6%) (Table 1). There were no significant differences in demographics across tertiles of dietary folate intake. When looking at diet composition, women in the highest tertile of dietary folate intake had significantly higher total calorie, dietary fiber, B vitamins, selenium, magnesium, calcium, potassium, vitamin C, vitamin E, and iron intake. Women with anovulatory cycles were more likely to be of younger age and nulliparous (Table S1).

**Table 1:** Demographic and Dietary Characteristics of BioCycle Women by Dietary Folate Intake.

	Total Cohort	Tertiles of Dietary Folate Equivalents (µg/day)			p-value^1^
Range of Intake (µg/day) (median)	77.3–2334.1 (445.9)	77.3–388.5 (325.0)	388.6–548.5 (444.9)	548.6–2334.1 (682.0)	
*n* (number of cycles)	509	169	170	170	
**Demographics** 2					
Age, yrs	27.5 (8.2)	27.3 (8.2)	27.5 (8.5)	27.3 (8.0)	0.94
BMI, kg/m^2^	24.1 (3.9)	24.3 (3.8)	24.3 (3.9)	23.6 (3.8)	0.16
Physical Activity, n (%)					0.46
Low	48 (9.5)	20 (11.9)	12 (7.1)	16 (9.5)	
Moderate	182 (36.0)	58 (34.5)	68 (40.0)	56 (33.1)	
High	275 (54.5)	90 (53.6)	90 (52.9)	97 (57.4)	
Race, n (%)					0.55
Caucasian	300 (59.4)	91 (54.2)	101 (59.4)	109 (64.5)	
African-American	100 (19.8)	46 (27.4)	27 (15.9)	27 (16.0)	
Other	105 (20.8)	31 (18.5)	42 (24.7)	33 (19.5)	
Years of Education, n (%)					0.33
≤ High School	65 (12.9)	25 (14.9)	21 (12.4)	18 (10.7)	
Post-Secondary	440 (87.1)	143 (85.1)	149 (87.7)	151 (89.4)	
Smoking Status, n (%)					0.72
Never or Former	487 (96.1)	160 (95.2)	164 (96.5)	163 (96.5)	
Current	20 (3.9)	8 (4.8)	6 (3.5)	6 (3.6)	
Nulliparous, n (%)	367 (73.6)	126 (77.3)	121 (72.9)	120 (70.6)	0.26
Age at Menarche, yrs	12.4 (1.2)	12.3 (1.3)	12.6 (1.3)	12.5 (1.1)	0.19
Cycle Length, days	28.8 (4.1)	28.8 (4.7)	28.9 (3.6)	28.8 (4.0)	0.87
**Dietary Nutrients** 2					
Total Calories, kcal/day	1608.1 (405.0)	1383.4 (322.1)	1593.4 (316.7)	1846.2 (426.6)	<0.0001
Total Fat, %	33.9 (6.3)	35.6 (6.6)	33.6 (5.6)	32.4 (6.3)	<0.0001
Carbohydrate, %	50.9 (8.2)	48.7 (8.5)	51.1 (7.5)	52.8 (8.2)	<0.0001
Protein, %	15.7 (3.4)	16.1 (3.7)	15.8 (3.5)	15.4 (3.0)	0.07
Cholesterol, mg/dL	209.4 (115.7)	197.1 (100.6)	209.5 (106.2)	221.6 (136.4)	0.0002
Total Fiber, g/day	13.6 (6.0)	10.5 (4.7)	13.3 (4.7)	16.9 (6.5)	<0.0001
Folate Equivalents, µg/day	368.9 (148.2)	309.6 (62.1)	455.7 (43.3)	735.3 (207.2)	<0.0001
Thiamin (B1), mg/day	1.4 (0.5)	1.0 (0.2)	1.3 (0.2)	1.8 (0.5)	<0.0001
Riboflavin (B2), mg/day	1.7 (0.6)	1.3 (0.4)	1.6 (0.4)	2.1 (0.7)	<0.0001
Niacin (B3), mg/day	19.5 (6.7)	15.6 (4.5)	19.0 (5.2)	24.0 (7.2)	<0.0001
Vitamin B6, mg/day	1.5 (0.6)	1.1 (0.3)	1.4 (0.4)	1.9 (0.6)	<0.0001
Vitamin B12, mg/day	4.2 (6.4)	2.9 (3.6)	3.9 (6.2)	5.7 (8.2)	0.37
Selenium, µg/day	88.4 (27.9)	74.4 (23.6)	88.7 (23.5)	101.9 (29.3)	0.003
Magnesium, mg/day	222.1 (74.3)	182.2 (61.2)	216.0 (57.6)	267.9 (76.1)	<0.0001
Calcium, mg/day	697.9 (281.9)	548.5 (194.1)	687.8 (232.7)	856.7 (315.3)	<0.0001
Potassium, mg/day	1917.4 (607.0)	1609.4 (460.0)	1931.6 (559.1)	2209.4 (635.6)	0.006
Caffeine, mg/day	92.1 (98.9)	85.6 (86.8)	95.1 (105.6)	95.5 (103.5)	0.15
Vitamin C, mg/day	70.0 (43.1)	54.0 (37.0)	71.3 (39.0)	84.6 (47.0)	0.0002
Vitamin E, mg/day	9.8 (7.2)	7.1 (3.6)	8.5 (3.7)	13.8 (10.1)	<0.0001
Iron, mg/day	12.3 (5.1)	8.6 (2.1)	11.5 (2.8)	16.8 (5.5)	<0.0001

1 Two-sided *P*-values were calculated using generalized linear mixed models. All comparisons take repeated measures and correlations between cycles into account. Dietary nutrients except for total calories and % carbohydrate, protein, and fat from calories are adjusted for total calorie intake.

2 Unless otherwise stated, values presented are mean (standard deviation).

3 Parity totals do not add up to 509 cycles due to 10 missing responses.

BioCycle participants had a mean dietary folate intake of 500.5 µg/day (range, 77.3–2334.1 µg/day) (Table 2). The mean was slightly lower than that reported in a similar age group of women in NHANES III, 718 µg/day, most likely due to the fact that BioCycle women were enrolled based on reporting currently not consuming multivitamins or supplements [25]. However, our mean folate intake was relatively higher compared to most European cohorts of reproductive age women, not reporting supplement use, most likely due to the mandated fortification of grains with folic acid in the US [26], [27]. On average, the dietary folate consumed by women in the BioCycle Study was 50.8% synthetic (folic acid) and 49.2% natural. Specifically, 29.1% of dietary folate came from fortified cereals, 41.1% from fortified grain products, 18.1% from vegetables, and 11.7% from beans.

**Table 2:** Intake of Folate by Type and Source in Women of the BioCycle Study.

Types of Folate(µg/day)	Mean	SD	Min	Median	Max	% Total Folate
Total Folate	368.9	148.2	69.2	336.4	1592.5	n/a
Folate Equivalents	500.5	217.8	77.3	445.9	2334.1	n/a
Synthetic Folate	187.5	111.6	10.1	158.1	1057.5	50.8
Cereal Folate	104.0	132.9	0.0	53.8	1120.3	29.1
Grain Folate	147.2	69.4	0.0	139.0	484.4	41.1
Natural Folate	181.7	82.2	49.2	166.2	954.3	49.2
Vegetable Folate	64.9	69.4	1.9	56.1	278.8	18.1
Bean Folate	42.0	87.3	0.0	0.0	716.8	11.7

Intake of total folate (in µg/day DFE) was not significantly associated with estradiol, LH, or FSH levels; however it was marginally associated with luteal progesterone levels (*P* for trend 0.08) (Table 3). Women in the highest tertile of synthetic folate intake had 16% (95% CI, 1–34%) higher luteal progesterone levels compared to women in the lowest tertile of synthetic folate intake (Table 3). When considering the source of synthetic folate, both grain and cereal folate did not have significant associations with progesterone. No type of dietary folate was significantly associated with estradiol, LH, or FSH.

**Table 3:** Association between dietary folate intake and estradiol and progesterone in the BioCycle Study.

		Estradiol			Progesterone		
Types of Folate (µg/day)	Tertile (Median)	% Change^1^	95% CI	*P* for trend	% Change^1^	95% CI	*P* for trend
Folate Equivalents	1 (325.0)	REF	REF	0.97	REF	REF	0.08
	2 (444.9)	−2.68	(−12.98, 8.85)		6.38	(−8.54, 23.73)	
	3 (682.0)	0.65	(−8.43, 10.63)		16.03	(−2.62 38.25)	
Synthetic Folate	1 (100.9)	REF	REF	0.56	REF	REF	0.05
	2 (157.9)	−6.09	(−16.45, 5.55)		12.01	(−4.03, 30.74)	
	3 (270.6)	2.33	(−7.04, 12.64)		15.70	(0.05, 33.80)	
Grain Folate	1 (83.2)	REF	REF	0.89	REF	REF	0.33
	2 (138.7)	4.79	(−3.10, 13.31)		5.76	(−5.79, 18.73)	
	3 (201.9)	3.04	(−8.08, 15.51)		4.66	(−7.00, 17.78)	
Cereal Folate	1 (14.0)	REF	REF	0.58	REF	REF	0.66
	2 (53.7)	−1.58	(−11.05, 8.89)		1.69	(−8.91, 13.51)	
	3 (192.4)	3.73	(−2.78, 10.68)		1.71	(−11.23, 16.53)	
Natural Folate	1 (116.1)	REF	REF	0.24	REF	REF	0.54
	2 (166.1)	−13.55	(−24.37, −1.18)		7.08	(−7.24, 23.61)	
	3 (242.0)	−13.74	(−29.37, 5.34)		14.21	(−1.85, 32.88)	
Vegetable Folate	1 (28.4)	REF	REF	0.89	REF	REF	0.55
	2 (56.0)	1.23	(−4.40, 7.20)		6.58	(−5.98, 20.82)	
	3 (101.5)	−1.65	(−7.13, 4.14)		5.87	(−8.54, 22.55)	
Bean Folate^2^	1 (0.0)	REF	REF	0.55	REF	REF	0.68
	2 (48.8)	27.27	(3.07, 57.15)		9.37	(−25.37, 60.27)	
	3 (160.4)	28.03	−(0.20, 64.25)		8.72	(−20.89, 49.41)	

Results for luteinizing hormone and follicle-stimulating hormone are not presented due to nonsignificant results.

1 Results of weighted linear mixed models adjusted for age (continuous), race (White, Black, Other), BMI (continuous), mean total calorie (continuous), fiber intake (continuous), and other reproductive hormones (log-transformed, continuous) by inverse probability weights. Results are expressed as % change, (exp(β)−1)×100.

2 Due to the low intake of bean folate, intake was categorized into non-consumers and consumers above and below the median intake of consumers (81.4 mcg/day).

Although intake of total folate was not significantly associated odds of anovulation, synthetic folate was significantly and inversely associated with the odds of anovulation (Table 4). Women in the 2^nd^ and 3^rd^ tertile of intake had 22% (95% CI −45%–68%) and 64% (95% CI 8–965%) decreased odds of anovulation respectively, compared to women in the 1^st^ tertile (*P* for trend 0.03). Intake of folate from fortified grain products and cereals (which is predominately synthetic folate) had similar associations for anovulation across tertiles. Women in the second category of bean folate (consumers of 0.1–81.4 mcg/day) had significantly decreased odds of anovulation compared to non-consumers; however there was no linear trend across tertiles.

**Table 4:** Association between dietary folate in tertiles and sporadic anovulation in the BioCycle study.

Types of Folate (µg/day)	Tertile (Median)	AdjustedOdds Ratio^1^	95% CI	*P* for trend
Folate Equivalents	1 (325.0)	REF	REF	0.12
	2 (444.9)	0.79	(0.34, 1.82)	
	3 (682.0)	0.49	(0.19, 1.23)	
Synthetic Folate	1 (100.9)	REF	REF	0.03
	2 (157.9)	0.68	(0.32, 1.45)	
	3 (270.6)	0.36	(0.14, 0.92)	
Grain Folate	1 (83.2)	REF	REF	0.01
	2 (138.7)	0.72	(0.32, 1.63)	
	3 (201.9)	0.33	(0.14, 0.81)	
Cereal Folate	1 (14.0)	REF	REF	0.07
	2 (53.7)	0.62	(0.30, 1.30)	
	3 (192.4)	0.44	(0.20, 0.96)	
Natural Folate	1 (116.1)	REF	REF	0.44
	2 (166.1)	1.46	(0.58, 3.70)	
	3 (242.0)	1.40	(0.48, 4.08)	
Vegetable Folate	1 (28.4)	REF	REF	0.99
	2 (56.0)	1.18	(0.50, 2.74)	
	3 (101.5)	1.03	(0.40, 2.64)	
Bean Folate^2^	1 (0.0)	REF	REF	0.68
	2 (48.8)	0.11	(0.01, 0.85)	
	3 (160.4)	1.24	(0.44, 3.47)	

1 Generalized linear mixed models adjusted for age (continuous), race (White, Black, Other), BMI (continuous), and mean total calorie and fiber intake (both continuous).

2 Due to the low intake of bean folate, intake was categorized into non-consumers and consumers above and below the median intake of consumers (81.4 mcg/day).

We assessed the effect of misclassification of anovulation through a sensitivity analysis by comparing the results of a commonly used classification for anovulation (progesterone ≤5 ng/mL) (n = 65 cycles) with a more conservative classification (progesterone ≤3 ng/mL, where long cycles with only one luteal phase progesterone measurement were classified as ovulatory) (n = 22 cycles). While the confidence intervals for the results were much wider and p-values were slightly higher when the most conservative classification was used due to decreased power, the direction and magnitude of the association of folate with anovulation was consistent. For example, the odds of anovulation in the third tertile of synthetic folate compared to the first tertile was 58% (95% CI 15–79%) decreased using the more liberal definition of anovulation (*P* for trend 0.02) and 73% (95% CI −37–95%) decreased using the more conservative definition of anovulation (*P* for trend 0.07).

There was no significant effect modification of the association between all types of folate and reproductive hormones by BMI (<25 vs. ≥25 kg/m^2^), smoking status (current vs. never or former), average alcohol intake (<2.7 (median) vs. ≥2.7 g/day), periodic high alcohol intake (<4 drinks vs. ≥4 drinks/day), or vitamin B12 intake (<3.1 (median) vs. ≥3.1 mg/day). The associations between types of folate and reproductive hormone levels were similar when restricting to ovulatory cycles and when using folate as a continuous exposure (data not shown).

## Discussion

We examined the relation of intakes of total folate and specific sources of folate with reproductive hormones and anovulation in a group of young, healthy women not using folate or other dietary supplements at enrollment. We found that while total folate intake (in dietary equivalents) was not significantly associated with reproductive hormone levels or anovulation, higher intake of dietary synthetic folate was significantly associated with higher luteal progesterone levels and decreased odds of anovulation. The observed association of high dietary synthetic folate intake with increased progesterone levels and decreased risk of anovulation highlights other potential beneficial effects of folic acid fortification that to date may not have been recognized.

To our knowledge this is the first study to look at the association between dietary folate and reproductive hormone levels and anovulation in humans; however, there have been similar studies looking at folic acid supplement use and ovulation in animals. In immature superovulated rats, it was shown that either an excess or deficiency of folates partially inhibited ovulation [9]. A later study in rhesus monkeys showed that a folate-restricted diet led to irregular menstrual cycles and progressively decreased pre-ovulatory serum estradiol and mid-luteal progesterone compared to monkeys fed a normal diet [28]. Finally, folic acid supplement use in pigs was shown to promote ovulation as documented by increased litter size [8].

Relevant studies in humans have largely focused on multivitamin supplement use and fertility but for the most part have found consistent results. In humans a significantly higher rate of conceptions was found after preconceptional multivitamin supplement use in comparison with a placebo-like trace element supplement use in a Hungarian randomized, double-blind, controlled trial (*n* = 7,905) [11]. Similarly, in a double blind, placebo-controlled study (*n* = 93) looking at the effects of FertilityBlend (a type of multivitamin) for women on fertility, it was found that after 3 months of supplement use, women on FertilityBlend had a trend toward increased mean mid-luteal progesterone, had significantly more days with luteal-phase basal temperatures over 98°F, and had a significantly higher rate of pregnancy than women in the placebo group [29]. Finally, a longitudinal cohort study (*n* = 18,555) found an inverse association between frequency of multivitamin use and ovulatory infertility [12]. While all of these studies are consistent with our findings of folic acid being beneficial for ovulation, none were able to pinpoint a nutrient that was responsible for this association.

The observed relation between synthetic folate and anovulation is biologically plausible. Specifically, folate deficiency and homocysteine accumulation can lead to reduced cell division, increased inflammatory cytokine production, altered nitric oxide metabolism, increased oxidative stress, elevated apoptosis, and disturbed methylation reactions [13]; all of which could subsequently affect oocyte development. Studies of oocyte quality in the in vitro fertilization setting have confirmed this showing that women receiving a folic acid supplement (and who subsequently had lower homocysteine follicular fluid concentrations) had better quality oocytes and a higher degree of mature oocytes compared to women who did not receive folic acid [30]. Although currently research is limited, it is possible that ovarian response to endogenous FSH pulses is also decreased in low folate conditions, which could lead to impaired ovulation. In women undergoing controlled hyperstimulation with recombinant FSH, carriers of the T allele in position 677 of the MTHFR gene (which leads to decreased enzyme activity and 5-methyltetrahydrofolate concentrations) have a decreased ovarian responsiveness to this hormone, fewer oocytes to be retrieved [31], and granulosa cells that produce less estradiol, basal and stimulated [32]. In regards to the association between higher intake of dietary folate and higher progesterone levels, while evidence in humans is limited, it has been shown that folate deficiency in the rhesus monkeys leads to depleted ovarian granulosa cells [28] and decreased levels of luteal progesterone [33]. The authors of these articles hypothesized that folate deficiency might led to an "ineffective" process or "abortive" attempt at cell reproduction, the functional consequences of which would be reflected in the reduced biosynthesis of the sex hormones in these germ cells [28].

The reason for our significant finding with synthetic folate as opposed to natural folate and anovulation could be partly due to the greater absorption rates of synthetic folate [34]. While calculating dietary folate equivalents attempted to address this issue, it is known to be an imperfect measure [35]. Natural food folate is present primarily in the reduced polyglutamated form while synthetic folic acid is a fully oxidized monoglutamate form of folate. Relative to folic acid, natural food folate has a lower proportion of folate that is absorbed and available for metabolic reactions and/or storage. Several luminal factors also seem to hinder the absorption of natural food folate including its limited release from the food matrix, destruction within the gastrointestinal tract, and incomplete hydrolysis of glutamates in excess of one [34]. Since consumption of folic acid fortified foods (such as ready to eat breakfast cereals) is associated with higher usual vitamin B12 intakes this could also lead to a higher bioavailability of synthetic folate [36]. Finally, it is possible that the presence of other micronutrients added to fortified foods could explain some of the association between synthetic folate and anovulation; however models, which adjusted for highly correlated dietary nutrients (such as vitamin B12) produced similar results.

The BioCycle Study had a number of strengths including the intensive monitoring of a large number of young, ethnically diverse women throughout 2 menstrual cycles, with multiple measures of reproductive hormones and dietary intake. While self-report of diet is subject to measurement error, the estimation of dietary intake with multiple 24-hour recalls (up to 4 per cycle) decreased the probability of dietary misclassification. Furthermore, a Danish validation study showed that 3 24-hr recalls over 4 weeks had a correlation of 0.49 with red blood cell folate (a biomarker of long-term folate status) [26]. The prospective design and exclusion criteria at baseline of the BioCycle Study strengthen the ability to draw inference, having reduced the potential for bias from known risk factors for anovulation. In addition, standardized assessment of a wide variety of participant and dietary characteristics increased the ability to adjust for confounding. Finally, since BioCycle participants were not taking supplements, this allowed us to examine the impact of low levels of folate intake from dietary sources alone.

Nevertheless, the study faced several limitations, including the small number of anovulatory cycles (*n = *42), which limited the power of our findings. However, even with this moderate number we observed a significant inverse association between synthetic folate intakes and odds of anovulation. In addition, women were only followed for 2 menstrual cycles. Since anovulation occurs commonly in many women, long-term findings may be quite different. In absence of a daily transvaginal ultrasound or daily first morning urine measurements, the direct detection of ovulation has some degree of misclassification. However, in a sensitivity analysis, the effect of synthetic folate on anovulation was strong and consistent regardless of the definition used for classification. While our study included the use of a fertility monitor to help time visits, bias could have been introduced through mistimed sample collection. However, various indicators of successfully timed visits were found to be unrelated to folate consumption, thus any misclassification is likely to be non-differential [37]. As this was an un-supplemented population, we were limited in evaluating the association of dietary folate and reproductive function at modest levels of consumption due to the small number of high consumers (>1000 μ/day). Given that this was an observational study, it is possible that residual confounding by other lifestyle factors that were poorly measured remains; however, adjustment for a variety of demographic and dietary characteristics had little impact on the results. Finally, since we restricted our study sample to healthy, regularly menstruating women, we limited the generalizability of our findings to other populations, specifically women who are known to be chronically anovulatory.

In conclusion, we observed that higher intake of dietary synthetic folate was significantly associated with higher luteal progesterone levels and decreased odds of anovulation. These results highlight the potential beneficial role of folic acid in reproductive outcomes such as sporadic anovulation among healthy women of reproductive age that to date might not have been fully recognized. Future studies are needed to confirm these findings as folic acid supplement use may have important implications in improving fertility outcomes.

## Supporting Information

Table S1 **Demographic and Dietary Characteristics of BioCycle Women by Ovulation Status.**
(DOCX)
