# Supplementary material for: The Impact of Dietary Folate Intake on Reproductive Function in Premenopausal Women: A Prospective Cohort Study
Source: PLoS One. 2012 Sep 26;7(9):e46276. doi: 10.1371/journal.pone.0046276 (PMC3458830; doi:10.1371/journal.pone.0046276)
Supplement: Table S1 — Demographic and Dietary Characteristics of BioCycle Women by Ovulation Status. (DOCX) [file pone.0046276.s001.docx]

Table S1. Demographic and Dietary Characteristics of BioCycle Women by Ovulation Status.

|  | Ovulatory | Anovulatory | p-value^1^ |
| --- | --- | --- | --- |
| *n* (number of cycles) | 467 | 42 |  |
| Age^2^, yrs | 27.9 (8.3) | 22.0 (5.3) | <0.001 |
| BMI, kg/m^2^ | 24.2 (3.9) | 23.1 (3.6) | 0.08 |
| Physical Activity, n (%) |  |  | 0.93 |
| Low | 44 (9.6) | 4 (8.7) |  |
| Medium | 168 (36.1) | 14 (33.3) |  |
| High | 253 (54.4) | 24 (57.1) |  |
| Race, n (%) |  |  | 0.93 |
| Caucasian | 276 (59.4) | 25 (59.5) |  |
| African-American | 91 (19.6) | 9 (21.4) |  |
| Other | 98 (21.1) | 8 (19.1) |  |
| Nulliparous^3^, n (%) | 327 (71.2) | 40 (100) | <0.001 |
| Cycle Length, days | 28.9 (4.0) | 27.8 (5.1) | 0.13 |
| Cycle Visits, n (%) |  |  | <0.001 |
| 5 | 2 (0.4) | 3 (7.1) |  |
| 6 | 15 (3.2) | 8 (19.1) |  |
| 7 | 102 (21.8) | 10 (23.8) |  |
| 8 | 348 (74.5) | 21 (50.0) |  |
| Total Calorie Intake, kcals/day | 1610.3 (399.1) | 1583.3 (470.7) | 0.74 |
| Dietary Fiber Intake, g/day | 13.4 (5.5) | 16.0 (9.6) | 0.03 |
| Dietary Folate Equivalents, µg/day | 503.2 (217.3) | 471.1 (223.1) | 0.44 |

^1^Two-sided *P*-values were calculated using generalized linear mixed models. All comparisons take repeated measures and correlations between cycles into account. Dietary nutrients except for total calories are adjusted for total calorie intake.

^2^Unless otherwise stated, values presented are mean (standard deviation).

^3^Parity totals do not add up to 509 cycles due to 10 missing responses.
